# Particle Swarm Optimized Fuzzy CNN With Quantitative Feature Fusion for Ultrasound Image Quality Identification

**DOI:** 10.1109/JTEHM.2022.3197923

**Published:** 2022-08-10

**Authors:** Muhammad Minoar Hossain, Md. Mahmodul Hasan, Md. Abdur Rahim, Mohammad Motiur Rahman, Mohammad Abu Yousuf, Samer Al-Ashhab, Hanan F. Akhdar, Salem A. Alyami, Akm Azad, Mohammad Ali Moni

**Affiliations:** Department of Computer Science and EngineeringMawlana Bhashani Science and Technology University 271385 Tangail 1902 Bangladesh; Institute of Information Technology, Jahangirnagar University 115523 Savar Dhaka 1342 Bangladesh; Department of Mathematics and StatisticsFaculty of Science, Imam Mohammad Ibn Saud Islamic University (IMSIU) Riyadh 13318 Saudi Arabia; Department of PhysicsFaculty of ScienceImam Mohammad Ibn Saud Islamic University (IMSIU) Riyadh 13318 Saudi Arabia; Faculty of Science, Engineering and TechnologySwinburne University of Technology Sydney Parramatta NSW 2150 Australia; School of Health and Rehabilitation SciencesThe University of Queensland 1974 Brisbane QLD 4072 Australia

**Keywords:** Ultrasound image, quantitative feature extraction machine (QFEM), particle swarm optimization (PSO), feature fusion, fuzzy convolutional neural network, feature extraction

## Abstract

Inherently ultrasound images are susceptible to noise which leads to several image quality issues. Hence, rating of an image’s quality is crucial since diagnosing diseases requires accurate and high-quality ultrasound images. This research presents an intelligent architecture to rate the quality of ultrasound images. The formulated image quality recognition approach fuses feature from a Fuzzy convolutional neural network (fuzzy CNN) and a handcrafted feature extraction method. We implement the fuzzy layer in between the last max pooling and the fully connected layer of the multiple state-of-the-art CNN models to handle the uncertainty of information. Moreover, the fuzzy CNN uses Particle swarm optimization (PSO) as an optimizer. In addition, a novel Quantitative feature extraction machine (QFEM) extracts hand-crafted features from ultrasound images. Next, the proposed method uses different classifiers to predict the image quality. The classifiers categories ultrasound images into four types (normal, noisy, blurry, and distorted) instead of binary classification into good or poor-quality images. The results of the proposed method exhibit a significant performance in accuracy (99.62%), precision (99.62%), recall (99.61%), and f1-score (99.61%). This method will assist a physician in automatically rating informative ultrasound images with steadfast operation in real-time medical diagnosis.

## I. Introduction

Ultrasound images have an immense impact in the medical diagnosis fields and other imaging fields. It is widely known that more than 25% of medical imaging diagnosis procedures are involved with ultrasound and some major imaging techniques MRI, x-rays, etc. are complemented by ultrasonic imaging [1]. Ultrasound images are formed from the scattered reflection waves which are formed with random energy and this causes generating speckle noise in images. It is inevitable to reduce preserved edges and speckle noise for diagnosis and interpretation of ultrasound images [2]. Most of the ultrasound image analysis and filtering methods concentrate on the effect of speckle noise and try to reduce its effects [3] but sometimes the noise makes images distorted, and blurred which impacts a significant effect on image quality. As ultrasound image plays a significant role in diagnosis, distorted images or other improper images may lead to inadequate information in the diagnosis system. The judgment of disease diagnosis based on ultrasound imaging depends on the dexterity of the physician. Uninformative ultrasound images open lead to making the wrong conclusion by the physician. Confirming the proper quality of an ultrasound image is a crucial step. Hence this research presents an approach to rate the quality of ultrasound images by sensing whether the image is normal, noisy, blurry, or distorted.

Different research has been induced to assess the quality of ultrasound images. On the base of ideal techniques and results, we have excerpted some such works in this paragraph. Rahman *et al.* [5] proposed an approach to determine the optimum threshold value of wavelet coefficient for the best speckle-noise reduction using Fisher discriminant analysis (FDA). As the main advantages, the authors claimed in terms of MSE, SNR, and EPF their proposed method effectively removed speckle noise from ultrasound images and provided better performances than other existing classical methods. Besides their method was more effective for highly inhomogeneous images. The novelty of this research was in the estimation of the threshold value. Authors Zhang *et al.* [29] presented a nonlinear diffusion method to remove the speckle noise from ultrasound images. The method was developed in the Laplacian pyramid domain. The principal advantage of this method was that it could remove speckle noise maximally by preserving small structures and edges of an ultrasound image. The prime novelty of this method was in the automatic identification of a gradient threshold for every pyramid layer of the nonlinear diffusion. Rahman *et al.* [7] presented an optimized speckle-noise reduction filter to reduce speckle noise from ultrasound images based on the differentiation of the diffusion in the direction of the gradient. The proposed method was compared with the existing Perona–Malik Filter Method in terms of some quantitative statistical measurements MSE, PSNR, RMSE, etc, and the method provided better results than the existing methods. The main advantages of this method included improving image quality while removing speckle noise as well as preserving and enhancing the edges of the image. The novelty of this method was in the capability of restoring fine details of an image. In the paper [9] an adaptive anisotropic diffusion technique was introduced for ultrasound images to reduce speckle noise. The prime advantage of this method was that it could remove noise from an ultrasound image with preserving edges by causing no blur between the frontiers of different regions. The novelty of this method was in the direction-oriented mechanism of speckle-noise reduction. The limitations of all methods discussed till now were that these methods could remove only speckle noise of ultrasound images. These methods couldn’t detect or mitigate other ultrasound image quality issues. Authors Singh *et al.* [10] mentioned the effectiveness of Local binary patterns (LBP) to measure the quality of synthetic ultrasound images. They also demonstrated the use of LBP in the analysis of texture features. The main advantage of this method was that the method could easily be used to generate the dataset of the synthetic ultrasound image. This method also developed an objective quality assessment for synthetic ultrasound images and this was the core novelty of this method. The whole experiment of this method was evaluated based on the feature of a single technique namely LBP and it was a fundamental limitation of this method.

All of the methods discussed earlier are the classical methods that perform quality analysis of ultrasound images by using various quantitative parameters like MSE, PSNR, etc. In recent years several intelligent works have been introduced for the exploration of ultrasound image quality based on artificial intelligence. The subsequent part of this paragraph presents some of these kinds of methods. Zhang *et al.* [4] presented a CNN-based image Quality assessment (IQA) model for ultrasound images. To establish the IQA model they had used a deep CNN and a residual network followed by a transfer learning approach. For evaluation of the IQA model two error metrics, LCC and SROCC had been used where PSNR and SSIM were used to evaluate the ultrasound images quality. Based on the result of these measurement metrics they had found that the CNN-based IQA model provided effective results. This model provided an automatic no-reference IQA based on Deep learning (DL) which was the prime advantage and novelty of this method. The proposed IQA technique had some subjective issues during image labeling and this was a fundamental limitation of this method. In the paper [6] a DL-based scheme FUIQA was introduced to assess the fetal ultrasound image quality with the realization of two DL models L-CNN and C-CNN. They had involved 8072 fetal abdominal images from approximately 492 ultrasound videos from which the model L-CNN localized the fetal abdominal region of interest (ROI) and C-CNN evaluated the ultrasound image quality based on that ROI. Later the results of the FUIQA scheme were assessed by three metrics ROI, SB, UV, and suggested that the local phase features were helpful to improve the performance of model L-CNN. An automatic quality control scheme for fetal ultrasound images was the main advantage and novelty of this method. However, the method was applicable only fetal ultrasound images which was a prime limitation of this method. Mostafiz *et al.* [8] proposed an automatic deep neural network system to detect and reduce speckle noise from ultrasound images. The coalescence of CNN and wavelet features had been used to detect and classify ultrasound images. They attained 98.54% accuracy, 98.19% sensitivity, and a specificity of 98.25% for image classification. They concluded that LDA in noise analysis shows better performance in terms of MSE, SNR, and EPF. This method could detect and remove speckle noise of an ultrasound image by itself which was the prime advantage and novelty of this method. This method was only applicable to the speckle noise of ultrasound images which was the main limitation of this method. A Machine learning (ML)-based scheme was developed in the paper [11] by using the AdaBoost algorithm, to measure the quality of fetal ultrasound images. The automated detection of stomach bubbles and the umbilical vein was also proposed with AdaBoost which takes less than 6 seconds. In the base of accuracy, specificity, sensitivity, and error results they had shown the detection of the stomach was more accurate than the umbilical vein. An intelligence scheme for fetal ultrasound image quality detection was the main advantage and novelty of this method. The whole research was designed based on only one dataset and only for fetal ultrasound images. These were the limitations of this method. All the existing research discussed till now either build techniques to reduce speckle noise or generates a quality assessment approach. However, all most every technique works with only certain types of ultrasound images such as speckle noise, fetal ultrasound image, etc. So, based on the correlation and analysis of previous works, this research decides to build an automatic quality rating scheme for multiple ultrasound quality issues.

The fundamental aim of this research is to rate whether an ultrasound image is normal, noisy, blurry, or distorted. To build the scheme this research performs feature fusion from an input ultrasound image by using DL and a customized feature extraction approach. After that classification is performed on these fused features to rate the quality of the input ultrasound image. The customized feature extraction technique of this research is named by Quantitative feature extraction machine (QFEM) and it extracts several quantitative features from an input ultrasound image. For DL-based feature extraction, this research modified the existing VGG-19 CNN model by adding a fuzzy layer to it. Two customary fuzzy operations namely fuzzification and defuzzification are utilized to construct the fuzzy layer. In any fuzzy process, fuzzification alters natural inputs to fuzzy states. After performing the fuzzy mechanism to those fuzzy states defuzzification alters the consequence in its natural form [30]. Fuzzification and defuzzification are the facile, operable, and feasible mechanisms that most researchers utilize to develop fuzzy logic design. For instance, the authors of the paper [31] developed a fuzzy scheme for analysis and matching the fingerprint. The authors of the paper [32] developed a convolution-based neuro-fuzzy architecture to do the analysis of sentiment from movie clips. In paper [33] the authors proposed a fuzzy CNN structure to predict traffic flow from precarious traffic accident data. In all of these methods [31], [32], the authors follow the approach of fuzzification and defuzzification in between isolated activities. The fuzzy VGG-19 CNN model of this research is optimized by utilizing the Particle swarm optimization (PSO) technique. PSO is a bio-inspired technique that finds an optimal solution from a solution space [13]. Nowadays PSO is widely utilized in various DL and ML approaches to increase the efficiency of models through the best optimization. For instance, the authors of the paper [34] utilized PSO to get the optimal parameters for CNN models. In the paper [35] the authors utilized PSO to optimize the parameters of the Support vector machine (SVM). Where, PSO increased the efficiency of SVM to classify different types of plants. The authors of papers [36], [37], [38] also utilized PSO to increase the efficiency of different DL techniques. Thus, this research decides to examine the efficiency of PSO. Following are the principal contributions involved in this research:

• This research increases the performance of the existing VGG-19 CNN model by adding a fuzzy layer with it and by optimizing the model using the PSO technique. Also using the fuzzy layer and PSO technique proposed method analysis the performance of different well-known CNN models.
• A fancy feature extraction technique named QFEM is presented in this research which performs excellent using only 120 features.
• The proposed method builds an automatic ultrasound image quality rating scheme with a low misdetection rate because of the fusion of features from two techniques QFEM and PSO optimized fuzzy VGG-19 CNN model.
• This research generates its own ultrasound image dataset of 2600 images to amplify the proposed scheme.

The next part of this paper is allocated in the following way: Section II describes the prime architecture of this research along with the related dataset. Section III presents the obtained results of this research with the necessary discussion. Finally, section IV concludes the overall work of this research.

## II. Materials and Methodology

**FIGURE 1.**
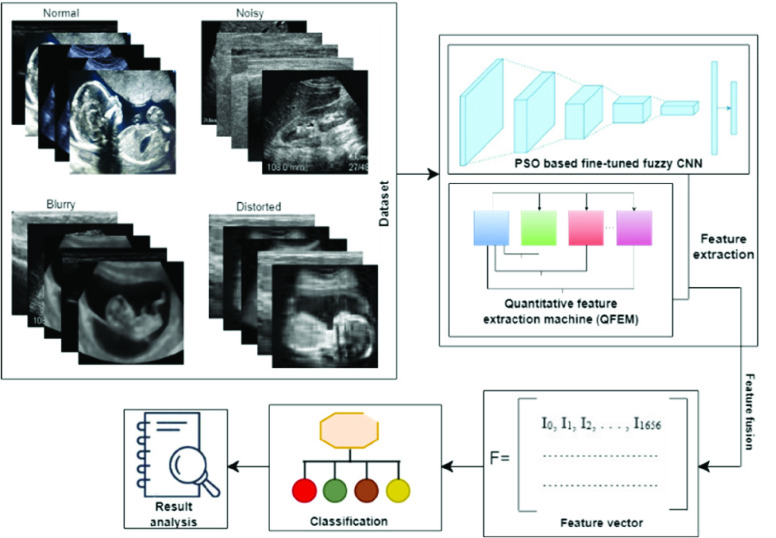
The fundamental architecture of this research.

This section presents the dataset and main formation of this research. Fig. 1 presents the overall formation of this research and section A to D narrates Fig. 1 in detail.

### A. Dataset

The dataset [49] of this research holds four types of ultrasound images namely normal, noisy, blurry, and distorted. Each type has a total of 650 ultrasound images. Thus, a total of 2600 images exist in the dataset. The images of the dataset are collected from various sources on the internet as well as from real-life diagnostic centers. TABLE 1 presents the sample ultrasound images for each class of mentioned dataset.

**TABLE 1:** Sample Images for Each Class of the Dataset

Class	Sample	
Normal		
Noisy		
Blurry		
Distorted		

### B. Feature Extraction

For feature extraction, this research uses both handcrafted and CNN features. Quantitative features are extracted by using handcrafted features and for this purpose, this research presents a novel feature extraction approach. For CNN-based feature extraction, the proposed method improves an existing CNN architecture by using an additional fuzzy layer with it. Section 1 and 2 describe the process of feature extraction in detail. Two popular feature selection techniques namely Minimum redundancy maximum relevance (mRMR) [44] and Recursive feature elimination (RFE) [45] are used to evaluate the redundancy of extracted features.

#### 1) Quantitative Feature Extraction Machine

**FIGURE 2.**
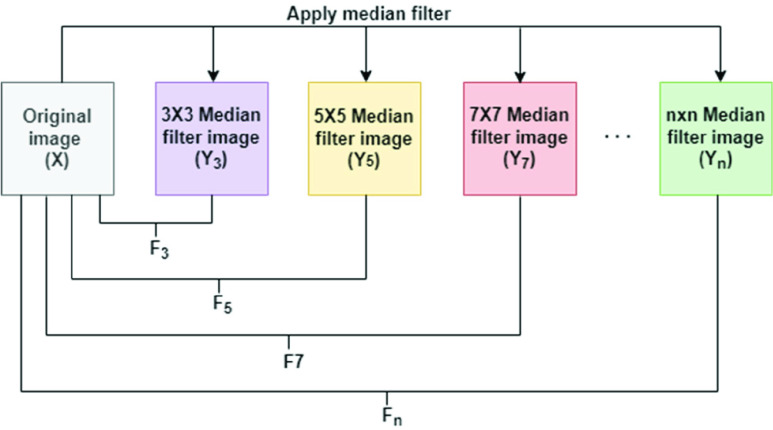
Structure of QFEM.

QFEM is a customized feature extraction approach presented in this research. QFEM aims to identify the pattern of quantitative features within the images to recognize the qualitative circumstance of that image. Fig. 2 illustrates the working method of QFEM at a glance. QFEM consists of N steps. In 1^st^ step of QFEM, an image (Y_3_) is gained from the input image (X) by using the median filter [12] with a $3\times 3$ convolution matrix. After that, 15 features are calculated from two images Y_3_ and X. These 15 features are identified as F_3_ in Fig. 2. TABLE 2 summarizes these features altogether. By analyzing several existing works (i.e- [40], [41], [42], [43]) on quantitative quality assessment metrics of images this research observes most of the methods commonly use these 15 features of TABLE 2. Hence these 15 features are selected in this research.

**TABLE 2:** Description of the Features of QFEM

Feature	Feature name	Calculation strategy
I_0_	Difference of mean (DM)	$DM = \mu (X)$- $\mu (Y_{n})$ Here, $\mu $ presents the mean value
I_1_	Difference of median (DMe)	$DMe = \tilde {M}(X) - \tilde {M}(Y_{n}))$ Here, $\tilde {M}$ presents the median value
I_2_	Mean Square Error (MSE)	$MSE=\frac {1}{mn}\sum \limits _{i = 0}^{m-1} \sum \limits _{j = 0}^{n-1} \left [{ X(i,j)-Y_{n}(i,j) }\right]^{2}$ Here, m and n present the height and width of the image, and Y_n_present the median filtered image with convolution matrix size ( $\text{n}\times \text{n}$)
I_3_	Difference of standard deviation (DSd)	$\sigma (S)=\sqrt {{\big[\sum \limits _{i = 1}^{N} \left ({s_{i}-\mu }\right)^{2}} \mathord {\Biggl /{ {\vphantom {{\big[\sum \limits _{i = 1}^{N} \left ({s_{i}-\mu }\right)^{2}} \left ({mn }\right)}} }\Biggl. } \left ({mn }\right)-1\big]};$ $DSd = \sigma \left ({X }\right)-\sigma (Y_{n})$ Here, ${\mathrm {N}} = mn$, and $\text{s}_{\mathbf {i}}$ is the i’th number of pixels of the image S
I_4_	Difference of Variance (DV)	$\sigma ^{2}\mathrm {(S)=}\sum \limits _{\mathrm {i = 1}}^{\mathrm {N}} \left ({s_{\mathrm {i}}\mathrm {-\mu } }\right)^{2} \mathrm {/}\left ({mn }\right)-1;$ ${\mathrm {DV}}= \sigma ^{2}\left ({X }\right)-\sigma ^{2}(Y_{n})$
I_5_	Peak Signal-to-Noise Ratio (PSNR)	$PSNR=\vert 10\log _{10}\frac {2^{n}-1}{MSE}\vert $
I_6_	Signal to Noise Ratio (SNR)	$SNR={10log}_{10}\frac {\sum \limits _{i = 0}^{m-1} \sum \limits _{j = 0}^{n-1} {[Y_{n}(i,j)] }^{2}}{\sum \limits _{i = 0}^{m-1} \sum \limits _{j = 0}^{n-1} {[X\left ({i,j }\right)-Y_{n}(i,j)] }^{2}}$
I_7_	Mean Absolute Error (MAE)	$\mathrm {MAE=}\frac {1}{\mathrm {mn}}\Sigma \left |{ \mathrm {X-}Y_{n} }\right |$
I_8_	Gradient magnitude similarity deviation (GMSD)	$\mathrm {w}\left ({\mathrm {k} }\right)=\frac {2\mathrm {G}_{\mathrm {x}}\left ({\mathrm {k} }\right)\mathrm {G}_{\mathrm {y}}\left ({\mathrm {k} }\right)\mathrm {+C}}{\mathrm {G}_{\mathrm {x}}^{2}\left ({\mathrm {k} }\right)+\mathrm {G}_{\mathrm {y}}^{2}\left ({\mathrm {k} }\right)\mathrm {+C}}\mathrm {;}GMSD=\sqrt {\frac {\sum \limits _{i=I}^{N} \left [{ w\left ({k }\right)-\left ({\frac {\sum \limits _{i = 1}^{N} {w\left ({k }\right)}}{mn} }\right) }\right]^{2}}{mn}} $, Here, G_x_(k) and G_y_(k) are the gradient magnitude at the position k for X and Y_c_ respectively. C is a constant and C > 0 and W(k) gradient magnitude similarity
I_9_	Structural similarity index measure (SSIM)	$SSIM\left ({X,Y }\right)=\vert \frac {\left ({2~\mu _{x}\mu _{y}+C_{1} }\right)\left ({2\sigma _{XY}+C_{2} }\right)}{\left ({\mu _{X}^{2}+\mu _{Y}^{2}+\mathrm {C} }\right)\left ({\sigma _{x}^{2}+\sigma _{y}^{2}+C_{2} }\right)}\vert;\,\, Here \,\,\, Y=Y_{n}$
I_10_	Covariance (Cov)	$Cov\left ({X,Y }\right)=\frac {\Sigma \left ({x_{i}-\mu _{x} }\right)\left ({y_{i}-\mu _{y} }\right)}{\mathrm {mn}}; \mathrm {Here Y=}\mathrm {Y}_{\mathrm {n}}$ Here, x_i_and y_i_ are the i’th numbers of pixels of the image X and Y_n_respectively.
I_11_	Difference of wavelet energy (DWE)	${\mathrm {E(S)}} = \frac {\sum \limits _{? = 1} {A_{i}^{2}+B_{i}^{2}} +C_{i}^{2}}{\mathrm {mn}}$; DWE = E(X) – E(Y_n_) Here, A_i_, $\text{B}_{\mathrm {i,}}$ and C_i_ presents the i’th number of pixels for 1-level wavelet subband images HL, LH, and HH respectively
I_12_	Difference of Skewness (DS)	$S_{k}(S)=\frac {\sum \limits _{i = 1}^{N} \left ({s_{i}-\mu }\right)^{3}}{mxn};$ DS = S_k_(X) – S_k_(Y_n_)
I_13_	Difference of Kurtosis (DK)	$K_{u}(S)=\frac {\sum \limits _{i = 1}^{N} \left ({x_{i}-\mu }\right)^{4}}{mxn};$ ${\mathrm {DK}} = K_{u}(X)$- $K_{u}(Y_{n})$
I_14_	Difference of Entropy (DE)	$H(S)=-\sum \limits _{i_{,}j = 1}^{N} {p\left ({\mathrm {i,}j }\right)\ast {log}_{2}\left ({p(i,j) }\right)};$ DE = H(X) - H(Y_n_) Here, p(i, j) is the probability of pixel (i, j)

**FIGURE 3.**
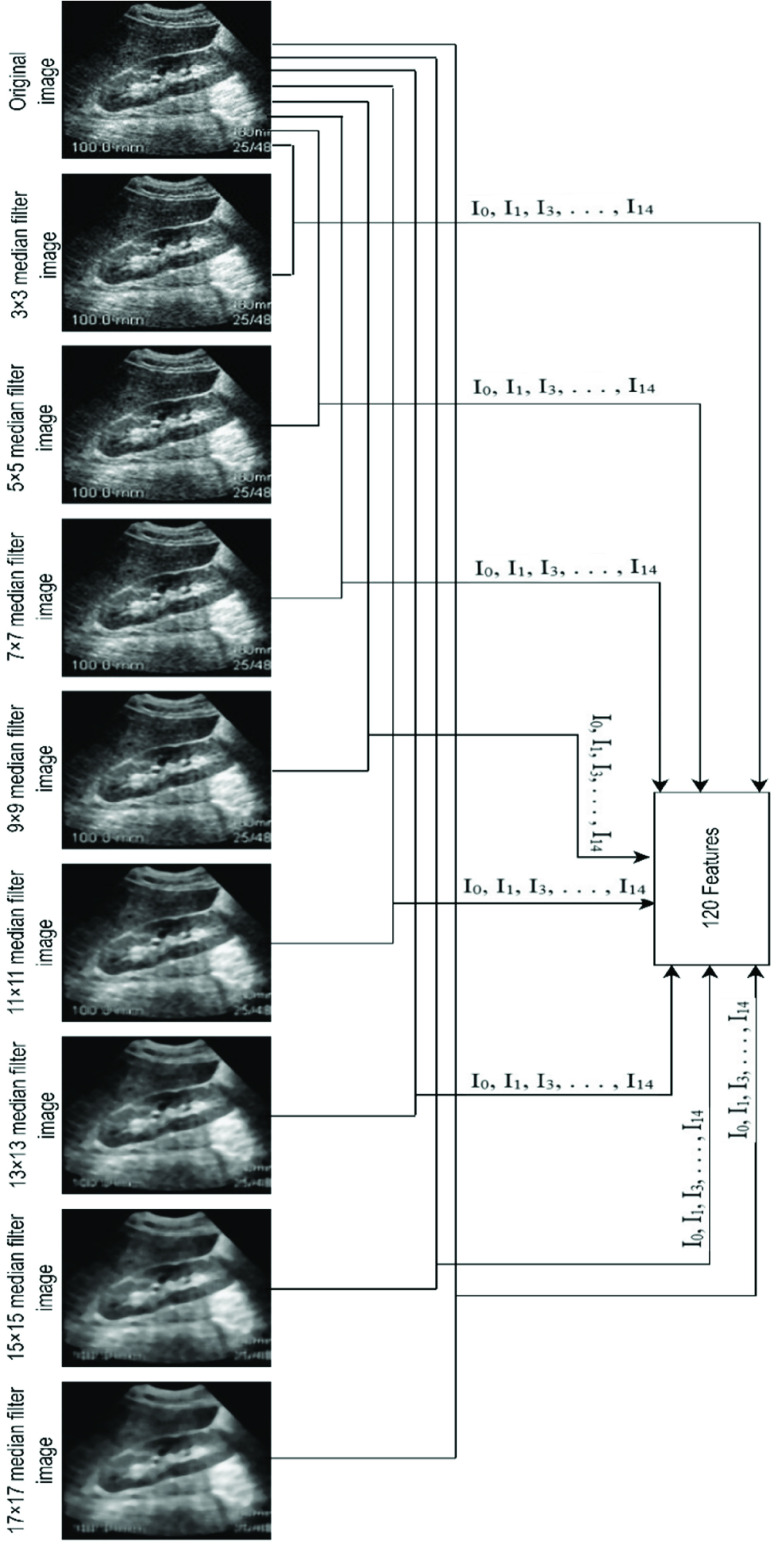
Mechanism of QFEM for a noisy image of the dataset.

**FIGURE 4.**
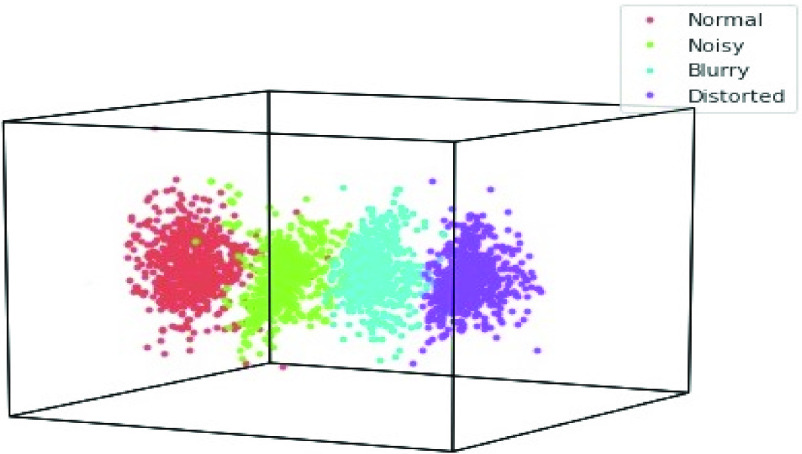
Cluster-wise visualization of the features of QFEM.

In the 2^nd^ step of QFEM, an image (Y_5_) is gained from the input image (X) by using the median filter with a $5\times 5$ convolution matrix. After that, 15 features (I_0_-I_14_) are calculated from two images Y_5_ and X. These 15 features are identified as F_5_ in Fig. 2. Sequentially median filter with $\text{n}\times \text{n}$ convolution matrix gives F_n_ from Y_n_ and X. After using QFEM with T number of steps there exists a total of $\text{T}\times 15$ features for an image. Although QFEM may apply with any number of steps, the number of steps should be ascertained according to user analysis. Because of getting optimum outcomes, this research uses 8-steps QFEM. For a noisy image of the dataset Fig. 3 demonstrates the mechanism of QFEM for this research. So, by QFEM a total of $8\times 15=120$ features gains in this research. Fig. 3 shows the cluster-wise visualization of these features for each class of the dataset. Fig. 4 shows that the features of QFEM provide a clear separability among classes.

The median filter can able to remove speckle noise from ultrasound images [39]. So, it can extract efficient features by using QFEM. Moreover, this research analyzes several filters (i.e-average filter, gaussian Filter, bilateral Filter, etc) as the core component of QFEM, and the median filter provide the supreme outcome over all of these. Thus, the median filter is an ideal suit for QFEM in this research. Algorithm 1 shows the working mechanism of QFEM.

Algorithm 1 **The Working Mechanism of QFEM**
Input: *2D image*
Output: *Feature vector*
*Initialization:*
1.
$n  =  2N-1, Where N  =  2, 3, 4, 5,...$
2.
$X \longleftarrow $ *Input image*
3.
$Y_{n} \longleftarrow $ *Apply median filter on* $X$ *using* $\text{n}\times \text{n}$ *kernel*
4.
$F_{v} \longleftarrow $ *Feature vector*
*Start:*
1. *For each* $N:$
2. *Find* $Y_{n}$
3. *Use (X, ${\text{Y}}_{n}$) to get $F_{n} \vert F_{n}$ {P_0_, $P_{1},\ldots, P_{14}$}*
4.
$F_{v}\longleftarrow F_{n}$
5. *end for*
6. *Show* $F_{v}$
*End:*

#### 2) PSO Based Fine-Tuned Fuzzy CNN

This research fine-tuned a pre-trained VGG-19 CNN model by adding an extra fuzzy layer with it. During fine-tuning PSO is used to optimize the hyperparameters of the model. PSO performs operation on a set of particle P = {p_1_, p_2_, $\text{p}_{3},\ldots,p_{\mathrm {i}}$}. At time t each p_i_ has a position x_i_ and a velocity v_i_. For the picked evaluation of objective function, the position is remembered by each $\text{p}_{\mathrm {i,}}$ and this information is stored by a memory pbest_i_. Memory pbest_i_is updated every time whenever p_i_finds a better position. Another memory gbest holds the best position at swarm level for any particle that has visited ever. PSO updates the value of x and v iteratively until an efficient solution is captured. Algorithm 2 presents the working mechanism of PSO.

Algorithm 2 **The Working Mechanism of PSO**
Input: *Set of random particles*
Output: *Best position of particles*
*Initialization:*
1. *Initialize random particle $P =$ { $p_{{\textit {1}}}$, $p_{{\textit {2}}}$, $p_{{\textit {3}}},\ldots, p_{i}$}.*
2.
$t \longleftarrow $ *Time*
3.
$\text{c}1 \longleftarrow $ *cognitive factors*
4. *c2 $\longleftarrow S$ocial factors*
5.
$u1, u2 \longleftarrow $ *Random values in the interval [0-1]*
6.
$w \longleftarrow $ *inertia weight*
7. *pbest $_{i} \longleftarrow $ best position of* $p_{i}$
8. *gbest $\longleftarrow $ global best position of particle*
*Start:*
1. *While (An efficient solution is not met)*
2. *For each $p_{i}$*
3. *Update velocity $v_{i} \vert v_{i}$(t+1) = $v_{i}$ (t)w+c1u1[ $pbest_{i}$- ${x} _{i}]+$ c2u2[gbest- $\text{x}_{i}]$*
4. *Update the position $x_{i} \vert x_{i}$(t+1)= $x_{i}(t)+v_{i}$(t+1)*
5. *Use objective function $f$ to evaluate the fitness value of $p_{i}$*
6. *Update $pbest_{i}(t) \vert pbest_{i}$(t+1)* $\begin{aligned} =\begin{cases} \displaystyle pbest_{i}\left ({t }\right) if f\left ({pbest_{i}\left ({t }\right) }\right)\le f(p_{i}\left ({t+1 }\right)) \\ \displaystyle (p_{i}\left ({t+1 }\right) if f\left ({pbest_{i}\left ({t }\right) }\right)>f(p_{i}\left ({t+1 }\right)) \end{cases} \end{aligned}$
7. *Update gbest(t) $\vert $ gbest(t+1) = max{f( $pbest_{i}\left ({t }\right))$, f(gbest(t))}*
8. *end for*
9. *end while*
*End:*

**FIGURE 5.**
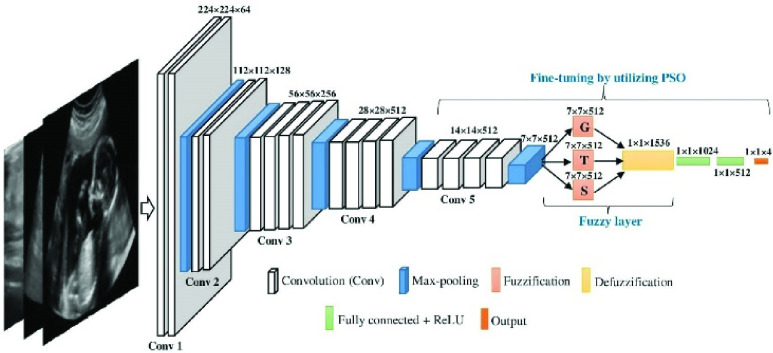
Illustration of proposed fuzzy layer-based VGG-19 fine-tuning by utilizing PSO.

Fig. 5 shows the architecture of the VGG-19 CNN model of this research. Like other CNN it has two parts namely feature extraction and feature classification part. The feature extraction part consists of a series of Convolution (Conv) layers including one max-pooling layer at the end of each Conv block and a fuzzy layer at the end of the last max-pooling layer. The rest of the network from the end of the fuzzy layer is defined as the feature classification part. The fuzzy layer is added in between the last max-pooling layer and the fully-connected layer as an additional layer with the existing VGG-19 structure. Two fundamental operations fuzzification and defuzzification are used to build the fuzzy layer. In the fuzzification stage, the output map of the last max-pooling layer is turned up to fuzzy maps by utilizing three membership functions namely Gaussian(G), Triangular(T), and S-shaped(S). For any value q in between p and r with a standard deviation $\sigma $, these functions can be defined as following way:

\begin{align*} G(\text {x};\text {q},\sigma)=&e^{-\frac {(x-q)^{2}}{2\sigma ^{2}}}\\ \mathrm {T}(\mathrm {x};\mathrm {p},\mathrm {r},\mathrm {q})=&\begin{cases} \displaystyle 0 & \mathrm {x}\le \mathrm {p}\\ \displaystyle \frac {x-p}{q-p}, & \mathrm {p} < \mathrm {x}\le \mathrm {q}\\ \displaystyle \frac {r-x}{r-q}, & \mathrm {q} < \mathrm {x} < \mathrm {r}\\ \displaystyle 0, & \mathrm {x}\ge \mathrm {r}\end{cases}\\ S(x,p,r)=&\begin{cases} \displaystyle 0, & x\le p\\ \displaystyle 2\left ({\frac {x-p}{r-p} }\right)^{2}, & p\le x\le \frac {p+r}{2}\\ \displaystyle 1-2\left ({\frac {x-r}{r-p} }\right)^{2}, & \frac {p+r}{2}\le x\le r\\ \displaystyle 1, & x\ge r\end{cases}\end{align*}

Different studies have found that the ReLU activation function with the highest value of six (6) helps the network learn the sparse features [46], [47]. Thus, we have selected the value of p and q based on the highest value (r_max_). The value of p was selected as half of r_max_, and q was selected as the sum of p with one-fourth of r_max_. In fuzzy logic, choosing membership functions is a non-trivial problem. The distribution of data is crucial in the selection process. Our research followed a trial-and-error process to choose the mentioned three membership functions. The cost of calculation and the number of parameters for membership functions have also been taken into account. The Gaussian membership function, for example, requires two parameters: mean and variance. It’s simpler to see the effect on inference when there are fewer parameters.

In the defuzzification stage each of the three fuzzy maps is turned to crisp values by using the Mean of max (M_m_) defuzzification technique. If x_j_ is the max possible degrees in any fuzzy map and N is the occurrence number of x_j_ then M_m_ can be defined as:

\begin{equation*} \mathrm {M}_{\mathrm {m}}=\frac {\sum \nolimits _{j=1}^{N}\bar {x_{j}}}{N}\end{equation*}

This research performs fine-tuning from Conv 5 block to the last output layer of the network given in Fig. 3. During finetuning, the hyperparameter of these layers is initialized as the random particles (P) for the PSO. After setting x_i_ and v_i_ for each p_i_, the proposed CNN model is executed for every p_i_ and during this execution, the value of x_i_, v_i_, pbest_i_ and gbest are updated for the gained results according to Algorithm 2. This execution performs iteratively and the parameter of gbest particle gained at final results is considered as the optimized parameter. After obtaining the optimum parameter proposed CNN model is used as a feature extractor by excluding the classifier part. TABLE 3 shows the general parameters of the PSO algorithm for this research.

**TABLE 3:** Parameters of PSO

Parameter	Value
Swarm size	10
iterations	15
Cognitive factor	1.4
Social factor	1.5
Inertia weight	0.9

To build the proposed CNN architecture this research uses the VGG19 CNN model. VGG19 is selected by analyzing several CNN architectures namely VGG19 [14], VGG16 [15], ResNet50 [16], InceptionV3 [17], Xception [18], and vanilla CNN baseline [48] because of providing contextual outcomes.

### C. Feature Vector

With QFEM this research extracts a total of 120 handcrafted features. From PSO-based fine-tuned fuzzy CNN this research extracts a total of 1536 features from the last fuzzy layer. Thus, by combining 120 and 1536 features a total of 1656 features exist in the feature vector for each image.

### D. Classification

Random forests (RF) algorithm is used to classify the feature vector. Once RF is trained the system can easily rate the quality of an ultrasound image. RF is a popular tree-based supervised ML algorithm that contains multiple Decision trees (DT) for classification tasks. It uses bagging ensemble [19] techniques which improves classification performance compared to other single classifiers. In the original RF model, the classification and regression trees algorithm are used which is a DT variant method that induces DT by recursive, top-down, greedy, and binary partitioning of the data set [20], [21]. In the paper [22] it is mentioned that a decision tree that contains N leaves partition the feature space into N no. of regions $\mathrm {R}_{\mathrm {n}}$, $\mathrm {1\le n\le N}$. So for each tree, the prediction function $\mathrm {f(x)}$ can be defined as

\begin{equation*} \mathrm {f}\left ({\mathrm {x} }\right)=\sum \limits _{\mathrm {n=1}}^{\mathrm {N}} \mathrm {C}_{\mathrm {n}} \mathrm {\pi \big(x,}\mathrm {R}_{\mathrm {n}}\big)\end{equation*}

where $C_{n}$ is a constant appropriate to n

\begin{align*} \pi \left ({\mathrm {x,}\mathrm {R}_{\mathrm {n}} }\right)=\begin{cases} \displaystyle 1, & \mathrm {if x\in R_{n}} \\ \displaystyle 0, & \mathrm {otherwise} \end{cases}\end{align*}

The RF is a robust method to handle noise and every DT of RF provides a unit result that assigns each input dataset to the most feasible label [23].

RF is used as a classifier in this research. Because of providing contextual outcomes, RF is selected in this work by analyzing several classifiers namely Logistic regression (LR) [24], Naive bayes (NB) [25], K-nearest neighbors (KNN) [26], and Extreme gradient boosting (XGB) [27].

## III. Result and Discussion

**FIGURE 6.**
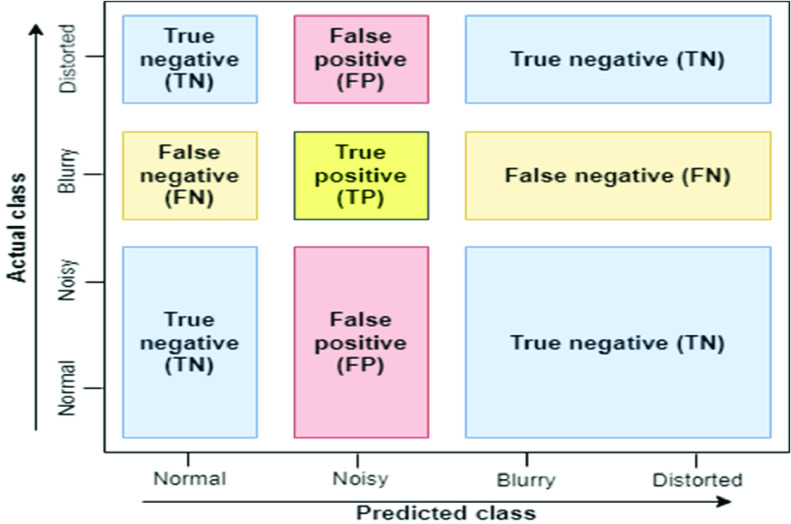
Demonstration of different parameters used to find performance measurement metrics.

The prime concern of this work is to develop an intelligent scheme for rating the quality of an ultrasound image. To develop the system the dataset of this research is partitioned in a ratio of 8:2, this ratio apprises that 80% of data are reserved for system training and the remaining 20% for system testing. All experiments of this work are examined by using 5-fold cross-validation [28] and usual performance measurement metrics of a classifier such as Precision, Recall, F1-score, Accuracy, as well as Normalize confusion matrix (NCM) are used to evaluate the efficiency of these experiments. TABLE 4 describes these metrics at a glance and Fig. 6 shows the demonstration of different parameters used to find these metrics.

**TABLE 4:** Description of Performance Measurement Metrics

Metrics	Description
Accuracy (A)	Presents the percentage of aggregated right prediction. $\text{A}= \frac {TP+TN}{FP+TP+FN+TN}\times 100$
Precision (P)	Defined as the measure of the quality of the model. P $= \frac {TP}{TP+FP}\times 100$
Recall (R)	Defined as the measure of the quantity of the model. ${\mathrm {R}} = \frac {TP}{TP+FN}\times 100$
F1-score (F1)	Presents how robust and precise a model is $\mathrm {F}1=2\times \frac {R\times P}{R+P}\times 100$
NCM	Presents actual and wrong detection rates of different classes in a tabular form.

To rate the image quality by using quantitative features this research presents the approach called QFEM. QFEM uses the median filter technique as a core component. Although any image filtering approach can be used to build QFEM the median filter is selected based on its suitable results by evaluating several filtering approaches. TABLE 5 shows the overall performance of QFEM for different filtering approaches. TABLE 5 shows the median filter holds the maximum accuracy of 97.69% whereas the Bilateral filter gives the nearest accuracy of 92.92%. TABLE 6 presents the fold-wise overall accuracy for the performance of TABLE 5. The exploration of TABLE 5 and VI prove that the median filter outperforms other filters in terms of performance for the dataset of this research.

**TABLE 5:** The Overall Performance of QFEM by Using Different Filtering Approaches

Filter	A	P	R	F1
Average	81.62	81.99	81.65	81.76
Gaussian	84.96	85.05	84.94	84.95
Min	72.85	72.76	72.94	72.80
Max	79.62	79.81	79.63	79.67
Bilateral	92.92	92.91	92.87	92.87
Median	97.69	97.69	97.66	97.67

**TABLE 6:** The Fold-Wise Overall Accuracy of QFEM by Using Different Filtering Approaches

Filter	Fold 1	Fold 2	Fold 3	Fold 4	Fold 5
Average	82.88	80.77	82.50	82.31	79.62
Gaussian	84.04	86.15	86.15	82.69	85.77
Min	71.15	72.50	72.50	75.38	72.69
Max	79.42	78.65	80.58	80.58	78.85
Bilateral	93.08	92.31	92.88	92.69	93.65
Median	98.27	96.92	97.69	97.31	98.27

TABLE 7 shows the overall performance of QFEM for this research (median filter as the core component) regarding features of different levels. TABLE 7 shows the overall accuracy of QFEM increased gradually till step 8. TABLE 8 presents the fold-wise overall accuracy for the performance of TABLE 7. TABLE 7 and 8 prove the justification for using 8 steps QFEM in this research.

**TABLE 7:** The Overall Performance of QFEM for Different Steps

Step No.	No. of feature	A	P	R	F1
1^st^	15	64.46	64.42	64.43	64.42
2^nd^	30	78.65	78.55	78.59	78.56
3^rd^	45	84.31	84.57	84.31	84.43
4^th^	60	87.38	87.72	87.39	87.52
5^th^	75	92.62	92.79	92.62	92.64
6^th^	90	93.15	93.25	93.17	93.20
7^th^	105	95.69	95.75	95.70	95.70
8^th^	120	97.69	97.69	97.66	97.67
9^th^	135	92.19	92.36	92.19	92.23
10^th^	150	84.65	84.67	84.64	84.64

**TABLE 8:** The Fold-Wise Overall Accuracy of QFEM for Different Steps

Step No.	Fold 1	Fold 2	Fold 3	Fold 4	Fold 5
1^st^	66.15	62.88	66.15	63.46	63.65
2^nd^	79.23	77.69	77.11	82.88	76.35
3^rd^	85.19	81.53	83.08	85.77	85.96
4^th^	87.31	87.50	86.35	89.23	86.54
5^th^	92.31	90.96	93.27	93.85	92.69
6^th^	93.08	91.35	94.04	93.46	93.85
7^th^	97.12	95.38	96.15	95.19	94.62
8^th^	98.27	96.92	97.69	97.31	98.27
9^th^	92.88	90.58	92.50	92.88	92.12
10^th^	83.85	85.96	86.92	82.11	84.42

This research fine-tuned several well-known CNN models. TABLE 9 presents the overall performance of these models. TABLE 10 presents the fold-wise overall accuracy for the performance of TABLE 9. Tables TABLE 9 and 10 present the overall performance of different CNN models without including fuzzy layer and PSO.

**TABLE 9:** The Overall Performance of Different Fine-Tuned CNN Models

CNN model	A	P	R	F1
VGG19	96.23	96.30	96.23	96.24
VGG16	93.15	93.28	93.17	93.20
ResNet50	94.31	94.39	94.32	94.34
InceptionV3	74.92	74.37	74.64	74.43
Xception	87.31	87.18	87.09	87.08
Vanilla	81.35	81.25	81.27	80.97

**TABLE 10:** The Fold-Wise Overall Accuracy of Different Fine-Tuned CNN Models

CNN model	Fold 1	Fold 2	Fold 3	Fold 4	Fold 5
VGG19	95.58	96.92	96.73	96.15	95.77
VGG16	94.62	92.12	92.50	92.69	93.85
ResNet50	94.23	93.08	94.62	95.19	94.42
InceptionV3	75.58	73.85	75.38	75.96	73.85
Xception	87.88	85.58	88.65	88.46	85.96
Vanilla	80.57	80.19	81.34	82.88	81.73

TABLE 11 presents the comparison among different CNN models and the proposed QFEM technique based on overall accuracy. Generally, handcrafted feature extraction gets lower performance than the DL-based approach but TABLE 11 shows that QFEM outperforms different CNN models. Hence, this research adds a fuzzy layer with different CNN models to improve their performance.

**TABLE 11:** Comparison Among Different CNN Models and Proposed QFEM Technique

QFEM	VGG19	VGG16	ResNet50	InceptionV3	Xception	Vanilla
97.69	96.23	93.15	94.31	74.92	87.31	81.35

TABLE 12 shows the overall performance of different fine-tuned CNN models including a fuzzy layer. TABLE 13 presents the fold-wise overall accuracy for the performance of TABLE 12.

**TABLE 12:** The Overall Performance of Different Fine-Tuned CNN Models With the Fuzzy Layer

CNN model	A	P	R	F1
VGG19	97.46	97.45	97.43	97.43
VGG16	95.81	95.85	95.81	95.82
ResNet50	96.04	96.07	96.05	96.05
InceptionV3	77.58	77.61	77.63	77.61
Xception	90.12	90.29	90.16	90.22
Vanilla	90.00	90.25	90.03	90.02

**TABLE 13:** The Fold-Wise Overall Accuracy of Different Fine-Tuned CNN Models With the Fuzzy Layer

CNN model	Fold 1	Fold 2	Fold 3	Fold 4	Fold 5
VGG19	98.46	96.54	96.73	98.08	97.50
VGG16	96.54	93.27	96.15	96.35	96.73
ResNet50	96.92	95.38	96.54	95.77	95.58
InceptionV3	77.88	75.96	77.12	76.73	80.19
Xception	90.77	87.88	90.19	90.38	91.35
Vanilla	89.42	89.23	90.00	90.38	90.96

TABLE 14 presents the comparison among different fine-tuned CNN models with and without using fuzzy layers. This comparison shows that the performance of CNN models improves because of the fuzzy layer. Where the fuzzy VGG-19 holds the max accuracy and TABLE 12 shows this accuracy is 97.46% which is less than the accuracy of 97.67% of QFEM. To further extend the performance of the proposed model, this research optimizes the fuzzy CNN models using PSO.

**TABLE 14:** Effect of the Performance of Different CNN Models Due to Fuzzy Layer and PSO

CNN model	VGG19	VGG16	ResNet50	InceptionV3	Xception	Vanilla
Original	96.23	93.15	94.31	74.92	87.31	81.35
With fuzzy layer	97.46	95.81	96.04	77.58	90.12	90.00
With fuzzy layer + PSO	98.38	97.73	97.85	79.23	91.23	94.73

TABLE 15 shows the overall performance of different PSO-based optimized fuzzy CNN models. TABLE 16 presents the fold-wise overall accuracy for the performance of TABLE 15. TABLE 15 and 16 present the efficiency of different fuzzy CNN models using PSO.

**TABLE 15:** The Overall Performance of Different Fine-Tuned Fuzzy CNN Models With PSO

CNN model	A	P	R	F1
VGG19	98.38	98.41	98.39	98.39
VGG16	97.73	97.76	97.72	97.74
ResNet50	97.85	97.87	97.85	97.86
InceptionV3	79.23	78.80	78.93	78.80
Xception	91.23	91.14	91.09	91.10
Vanilla	94.73	94.70	94.69	94.69

**TABLE 16:** The Fold-Wise Overall Accuracy of Different Fine-Tuned Fuzzy CNN Models With PSO

CNN model	Fold 1	Fold 2	Fold 3	Fold 4	Fold 5
VGG19	98.27	97.12	99.42	98.65	98.46
VGG16	97.88	97.50	97.69	98.65	96.92
ResNet50	98.46	97.31	97.50	97.88	98.08
InceptionV3	80.96	78.46	78.08	80.38	78.27
Xception	91.35	89.62	90.96	93.08	91.15
Vanilla	94.61	95.00	93.65	94.61	95.76

In terms of accuracy TABLE 14, shows the comparison among different fuzzy CNN models of this scheme with and without PSO. TABLE 14 presents VGG19 fuzzy CNN architecture with PSO holds the most compatible accuracy of 98.38%, which outperforms the performance of all individual techniques observed till now in our result and discussion part.

**FIGURE 7.**
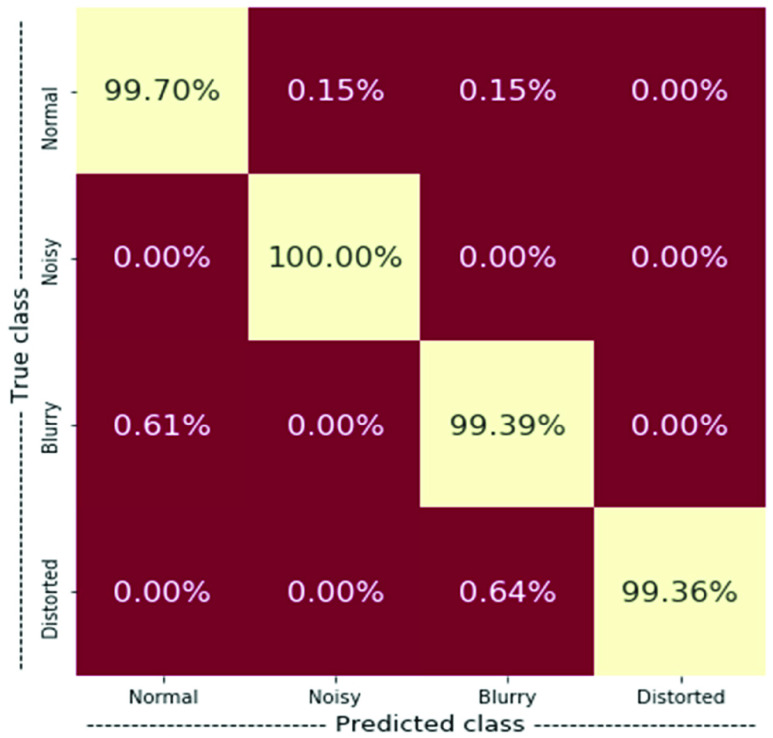
NCM of the proposed scheme.

The feature fusion of the QFEM and PSO-based fuzzy VGG19 model provides the actual outcome of this research and TABLE 17 presents this result. TABLE 18 presents the fold-wise accuracy for the result of TABLE 17. Fig.7 presents the NCM of this scheme for the result of TABLE 17.

**TABLE 17:** Performance of Proposed Scheme

Technique	Class	A	P	R	F1
QFEM + PSO based fuzzy VGG-19 + RF classifier	Normal	99.69	99.39	99.69	99.54
	Noisy	100	99.85	100	99.93
	Blurry	99.39	99.24	99.39	99.31
	Distorted	99.36	100	99.36	99.68
	Overall	99.62	99.62	99.61	99.61

**TABLE 18:** The Fold-Wise Accuracy of the Proposed Scheme

Fold 1	Fold 2	Fold 3	Fold 4	Fold 5
99.81	99.23	100	99.42	99.62

**FIGURE 8.**
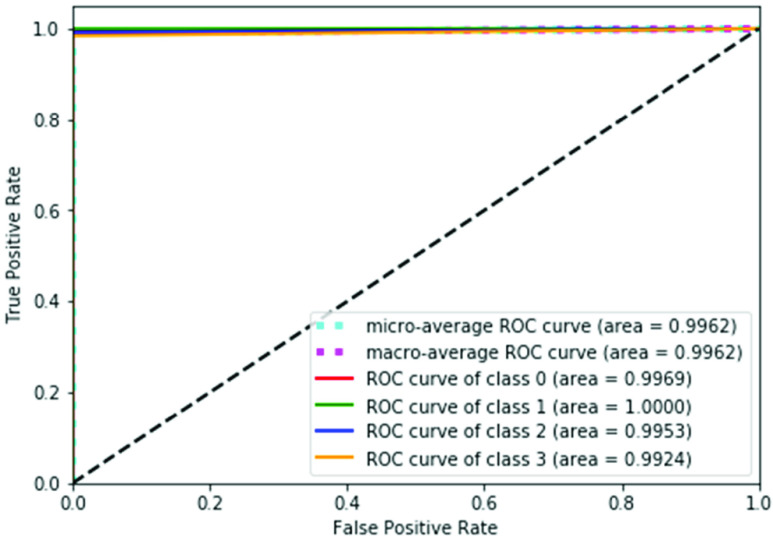
Roc curve of the proposed scheme, where classes 0,1,2 and 3 present Normal, Noisy, Blurry, and Distorted ultrasound images respectively.

The receiver operator characteristic (ROC) curve is a performance evaluation metric of a classifier. It presents the TP rate against the FP rate. In this curve, the more the value of the area under the curve is closer to one the more, good the classifier is. Fig. 8 shows the ROC curve for this research. TABLE 19 shows the performance comparison of this research with and without feature fusion. TABLE 19 shows that using QFEM and PSO-based fuzzy VGG19 CNN the highest accuracy gained is 97.67% and 98.38% respectively. Meanwhile, the fusion of these two techniques provides an overall accuracy of 99.62% which outperforms each individual technique.

**TABLE 19:** Comparison of the Performance of this Research With and Without Feature Fusion

	Technique	A	P	R	F1
Without fusion	QFEM	97.69	97.69	97.66	97.67
	PSO-based fuzzy VGG19	98.38	98.41	98.39	98.39
With fusion	QFEM + PSO-based fuzzy VGG19	99.62	99.62	99.61	99.61

This research analyzes several classifiers and from those RF is selected for giving the most preferable outcome. TABLE 20 presents the overall performance of different classifiers. TABLE 20 presents that the RF classifier provides the highest accuracy of 99.62%. TABLE 21 presents the fold-wise overall accuracy for the performance of TABLE 20. TABLE 20 and 21 prove the justification for utilizing RF in this scheme.

**TABLE 20:** The Overall Performance of Different Classifiers for the Proposed Scheme

Classifier	A	P	R	F1
LR	92.88	92.99	92.91	92.92
NB	87.85	87.71	87.86	87.66
KNN	61.58	59.05	61.06	59.73
XGB	99.04	99.03	99.03	99.03
RF	99.62	99.62	99.61	99.61

**TABLE 21:** The Fold-Wise Overall Accuracy of Different Classifiers for the Proposed Scheme

Classifier	Fold 1	Fold 2	Fold 3	Fold 4	Fold 5
LR	92.88	94.23	89.23	92.12	95.96
NB	87.69	86.54	88.46	89.04	87.50
KNN	61.73	60.77	60.58	64.42	60.38
XGB	100	98.27	98.85	99.42	98.65
RF	99.81	99.23	100	99.42	99.62

To evaluate the redundancy of the features, this research examines two feature selection techniques namely mRMR and RFE. TABLE 22 presents the overall performance of the proposed model for these techniques. TABLE 23 presents the fold-wise overall accuracy for the performance of TABLE 22. TABLE 22 presents that mRMR provides the best accuracy of 97.54% between mRMR and RFE and this result is less than the proposed model (99.62%). This indicates the 1656 features need no redundancy reduction.

**TABLE 22:** The Overall Performance of Different Feature Selection Techniques

Technique	A	P	R	F1
mRMR	97.62	97.59	97.60	97.58
RFE	95.15	95.13	95.12	95.10

**TABLE 23:** The Fold-Wise Overall Accuracy of Different Feature Selection Techniques

Technique	Fold 1	Fold 2	Fold 3	Fold 4	Fold 5
mRMR	97.88	96.34	97.69	97.88	98.26
RFE	94.61	95.38	94.80	95.19	95.76

From the analysis of related research as far as we know this is the first DL-based work to rate Ultrasound image quality. Hence this research puts no comparison with existing approaches to evaluate the performance of the proposed method.

## IV. Conclusion

This research presents an intelligent model to rate whether an Ultrasound image is normal, noisy, blurry, or distorted. To develop the scheme proposed method performs feature fusion from an ultrasound image by using a customized feature extraction approach and a PSO-based fuzzy VGG19 CNN technique and then the RF classifier recognize the quality type of that image from the fused features. Based on the results we have found the proposed approach as an efficient system for ultrasound quality rating by holding an inaccuracy of 0.38% only. In the future, besides the quality rating, we will try to restore the quality of an ultrasound image to normal if the quality is not detected as normal. However, the proposed method will assist physicians to make any decision during ultrasound imaging-based diagnosis.
